# Visual acuity time in range: a novel concept to describe consistency in treatment response in diabetic macular oedema

**DOI:** 10.1038/s41433-023-02507-x

**Published:** 2023-03-28

**Authors:** Igor Kozak, Ian Pearce, Chui Ming Gemmy Cheung, Tobias Machewitz, George N. Lambrou, Daniel Molina, Lima Suleiman, Hossam Youssef, Neil M. Bressler

**Affiliations:** 1 Moorfields Eye Hospitals UAE, Abu Dhabi, United Arab Emirates; 2 St Paul’s Eye Unit, Royal Liverpool University Hospital, Liverpool, UK; 3 Singapore National Eye Centre, Singapore Eye Research Institute, Singapore, Singapore; 4 Bayer AG, Berlin, Germany; 5 Bayer Consumer Care AG, Basel, Switzerland; 6 Bayer Middle East FZE, Dubai, United Arab Emirates; 7 Department of Ophthalmology, Johns Hopkins University School of Medicine, Baltimore, MD USA

**Keywords:** Outcomes research, Retinal diseases

## Abstract

**Objective:**

To assess ‘time in range’ as a novel measure of treatment response in diabetic macular oedema (DMO).

**Methods:**

This post hoc analysis of the Protocol T randomised clinical trial included 660 individuals with centre-involved DMO and best-corrected visual acuity (BCVA) letter score ≤78–≥24 (approximate Snellen equivalent 20/32–20/320). Study participants received intravitreal aflibercept 2.0 mg, repackaged (compounded) bevacizumab 1.25 mg, or ranibizumab 0.3 mg given up to every 4 weeks using defined retreatment criteria. Mean time in range was calculated using a BCVA letter score threshold of ≥69 (20/40 or better; minimum driving requirement in many regions), with sensitivity analyses using BCVA thresholds from 100 to 0 (20/10 to 20/800) in 1-letter increments.

**Results:**

Time in range was defined as either the absolute or relative duration above a predefined BCVA threshold, measured in weeks or as a percentage of time, respectively. Using a BCVA letter score threshold of ≥69 (20/40 or better), the least squares mean time in range (adjusted for baseline BCVA) in Year 1 was 41.2 weeks with intravitreal aflibercept, 4.0 weeks longer (95% CI: 1.7, 6.3; *p* = 0.002) than bevacizumab and 3.6 weeks longer (1.3, 5.9; *p* = 0.004) than ranibizumab. Overall, mean time in range was numerically longer for intravitreal aflibercept for all BCVA letter score thresholds between 92 and 30 (20/20 to 20/250). In the Day 365–728 analysis, time in range was 3.9 (1.3, 6.5) and 2.4 (0.0, 4.9) weeks longer with intravitreal aflibercept vs bevacizumab and vs ranibizumab (*p* = 0.011 and 0.106), respectively.

**Conclusion:**

BCVA time in range may represent another way to describe visual outcomes and potential impact on vision-related functions over time for patients with DMO and provide a better understanding, for physicians and patients, of the consistency of treatment efficacy.

## Introduction

In clinical trials, central visual acuity (VA) is a surrogate outcome for vision-related functions such as reading, driving, watching television, and recognising faces. There are some disadvantages of creating a binary outcome (for instance, whether patients gain or lose ≥15 Early Treatment Diabetic Retinopathy Study [ETDRS] letter scores [equivalent to 3 Snellen lines]) from a continuous variable such as VA [1]. Mean VA change (sometimes truncated to ±3 standard deviations to minimise the effects of outliers) from baseline to a particular timepoint offers advantages by simultaneously considering improvement and worsening, and has become a well-established endpoint with which to compare VA outcomes across study participants and treatment groups. Calculating mean change in VA from baseline, however, only reflects an assessment at a single point in time and is an average for a population (or individual): it does not determine the proportion of individuals in which a clinically relevant threshold (e.g. such as VA required for reading or driving) is achieved at various timepoints over the course of the disease. Mean change in VA from baseline at a particular timepoint also does not tell us anything about the duration of time spent over that clinically relevant threshold. Furthermore, average gain in VA from baseline at Year 1 does not mean that the benefit occurred consistently from Month 1 to Month 12. These considerations may be important when VA can fluctuate with recurrence of disease such as diabetic macular oedema (DMO), or when vision can fluctuate from a complication that waxes and wanes, such as vitreous haemorrhage that clears either spontaneously or following vitrectomy, or cataract that is cleared with surgery.

**Fig. 1:**
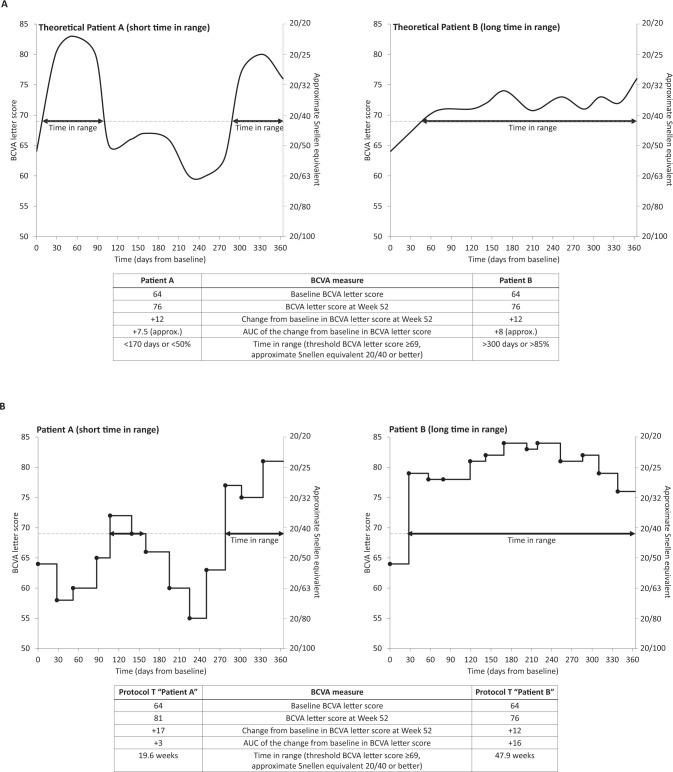
Calculating the time in range. Illustrative comparison between the AUC, time in range, and mean change in BCVA approaches when evaluating vision outcomes (**A**); the curves represent two different theoretical patients, each with the same baseline and endpoint BCVA, similar AUC, but differing durations of time in range above a BCVA threshold of ≥69 letters. Example of the calculation of different endpoints in two Protocol T Study participants over Year 1 (**B**). The AUC takes into account all of the changes relative to the baseline value and provides the average letter score gain across the entire treatment period. Time in range is shown along the horizontal axis and describes the amount of time a BCVA score is above a predetermined threshold level. AUC area under the curve, BCVA best-corrected visual acuity.

An area under the curve (AUC)-type analysis [2, 3] can be used to follow the study participant’s journey over time when disease recurrence or temporary fluctuations in vision may occur and have been calculated post hoc in the Diabetic Retinopathy Clinical Research (DRCR) Retina Network Protocol T [3–5]. However, AUC-type analyses do not fully capture variability in VA over time, as two different patients could have similar AUC of change in best-corrected visual acuity (BCVA) but spend markedly different amounts of time above a particular BCVA threshold (Fig. 1A).

Time in range is an alternative, or additional, approach to describe BCVA fluctuations reflecting the dynamics of a retinal disease such as DMO. The concept of time in range is adapted from its use in the evaluation of glucose control in diabetes [6, 7], applying the concept in DMO to evaluate the time that study participants spend at or above a BCVA threshold that is judged to be clinically relevant. The primary aim of this post hoc analysis using publicly available data from the DRCR Retina Network Protocol T is to introduce the concept of time in range as a clinically relevant functional endpoint for ophthalmologic indications.

## Subjects and methods

### Protocol T study

This post hoc analysis utilises data from the DRCR Retina Network Protocol T trial (NCT01627249). The study was chosen as it was a large, well-designed trial for which data were available [8], but the analyses and conclusions presented herein are solely the responsibility of the authors and have not been reviewed or approved by DRCR Retina Network.

A detailed description of the methodology, participants, and outcomes has been published [4] and the complete protocol is available online [8]. The Protocol T study adhered to the Declaration of Helsinki guidelines and was approved by institutional review boards at all sites. Study participants provided written informed consent. In brief, participants with a BCVA ETDRS letter score ≤78 (approximate Snellen equivalent 20/32 or worse) and ≥24 (approximate Snellen equivalent 20/320 or better) were randomly assigned (1:1:1) to be treated with intravitreal aflibercept (IVT-AFL) 2 mg, repackaged (compounded) bevacizumab (IVT-BEV) 1.25 mg, or ranibizumab (IVT-RAN) 0.3 mg, injected into the study eye at baseline and every 4 weeks thereafter unless BCVA was ≥84 (approximate Snellen equivalent 20/20 or better) with an optical coherence tomography (OCT) central subfield thickness (CST) <250 µm on Zeiss Stratus (<320 if male or <305 if female on Heidelberg Spectralis; <305 or <290, respectively, on Zeiss Cirrus) and there was no improvement or worsening in response to the past two injections. Improvement/worsening was defined as an increase/decrease in VA of ≥5 letters (1 line) or a decrease/increase in CST ≥10%.

Starting at the 24-week visit, irrespective of VA and CST, an injection was withheld if there was no improvement or worsening on both VA and CST measurements after two consecutive injections, but reinitiated if the VA letter score or CST worsened due to DMO. This strict retreatment protocol allowed flexible individualised treatment, as needed, to maximise gains in BCVA for all participants. During the first year, follow-up visits occurred every 4 weeks (±1 week). During the second year, visits were every 4–16 weeks, depending on treatment course.

The prespecified primary outcome measures were change in VA, assessed by electronic ETDRS letter scores, from baseline to 12 months/24 months in the overall population and cohorts of participants with a baseline VA letter score of 78‒69 (between 20/32 and 20/40) or <69 (20/40 or worse).

### Time-in-range post hoc analysis

The time-in-range calculation concept is shown in Fig. 1B. Individual variability in BCVA over 1 year is shown for two participants enrolled in the Protocol T study. Both had a similar BCVA at baseline and mean change in BCVA by the end of Year 1; however, one participant had substantial variability in BCVA over time. Applying a BCVA threshold (letter score of ≥69; approximate Snellen equivalent 20/40, corresponding with the legal limit of VA in the better-seeing eye required to drive a motor vehicle in many countries [9]), there is a clear difference between the two individuals in the time spent above the threshold—their BCVA time in range, or “time spent 20/40 or better” for this example.

Mean time in range (in weeks) was calculated for a BCVA letter score threshold of ≥69; approximate Snellen equivalent 20/40 (primary analysis) and, in sensitivity analyses, for all thresholds from 100 to 0 (in 1-letter increments), an approximate Snellen equivalent of 20/10 to 20/800, using all BCVA data available over Day 0–364, 365–728, or 0–728, whereby participants were assumed to have the same BCVA value until a newer measurement was performed. Protocol T data through Year 1 were analysed for the primary outcomes of this analysis. Two-year outcomes are provided as supplemental analyses, as more limitations must be considered for the data between 1 and 2 years given the variable follow-up schedule.

To determine the effect of the BCVA threshold applied and the effect of the participants’ baseline BCVA on the time-in-range outcome, time in range for the letter score threshold of ≥69 (20/40 or better) and also of ≥59 and ≥79 (20/32 and 20/25 or better) were calculated for the overall population, as well as according to and adjusted for baseline BCVA.

Furthermore, in a ‘responder-type’ analysis, the proportion of participants above BCVA letter score threshold of ≥69 (approximate Snellen equivalent 20/40 or better), the “responder threshold” agreed a priori, was calculated for time in range of 0–100% (in 1% increments).

The number of BCVA assessments in Year 1 (Day 0–364) and Year 2 (Day 365–728) of Protocol T (Supplementary Table 1) were analysed to identify participants who had a sufficient number of BCVA assessments to justify a time-in-range analysis; ≥7 BCVA assessments over the first year and ≥4 assessments over the second year were chosen as the thresholds, as these cut-offs, which corresponded with values at least every 2 months in Year 1 and at least every 3 months on Year 2, were deemed to provide appropriate datapoints with which to calculate time in range with sufficient accuracy. Thus, of the 660 eyes included in the original study, 22 eyes were excluded from the Day 0–364 analysis because they had <7 BCVA assessments and 85 eyes were excluded from the Day 0–728 and Day 365–728 analyses because participants had <4 assessments within Year 2.

### Statistical analyses

Baseline BCVA-adjusted analysis of covariance (ANCOVA) was used to test the least squares mean differences in the time in range between the three anti–vascular endothelial growth factor (VEGF) agents. The model assumed that the residuals (the error between the prediction of the model for every participant and the real time in range) were normally distributed; visual diagnostic plots were performed to confirm the normality of the residuals of the ANCOVA model. The *p* values were not meant to test statistical significance, as analyses were performed post hoc. No extreme values/outliers were excluded from, or truncated for, the analyses. All *p* values were two-sided; there were no adjustments to *p* values for multiple comparisons.

## Results

Participant baseline demographics and disease characteristics, and participant disposition in Protocol T have previously been described [4]. Of the overall population, 638/660 (97.6%) participants had ≥7 BCVA assessments over Day 0–364, and 575/660 (87.1%) had both ≥7 BCVA assessments over Day 0–364 and ≥4 assessments over Day 365–728, and both groups were included in the analysis. Baseline demographics and disease characteristics (Supplementary Table 2) were consistent with the full Protocol T population [4], with mean BCVA letter scores across the three treatment groups of 64.5–65.1 (approximate Snellen equivalent 20/50).

**Fig. 2:**
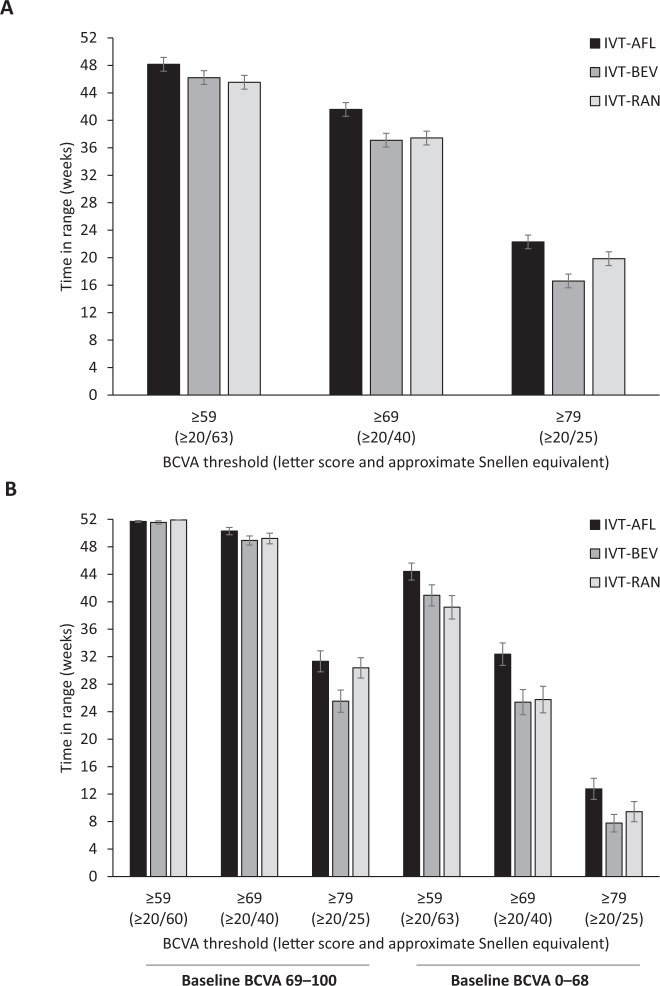
Mean time in range for intravitreal aflibercept, bevacizumab, and ranibizumab according to categorical BCVA letter score thresholds. Mean (±SEM) time in range for intravitreal aflibercept, bevacizumab, and ranibizumab according to BCVA letter score thresholds of ≥59, ≥69, and ≥79 (approximate Snellen equivalents of 20/63, 20/40, and 20/25 or better, respectively) in the Day 0–364 analysis (**A**) for the overall population and (**B**) according to baseline BCVA. BCVA best-corrected visual acuity, IVT-AFL intravitreal aflibercept, IVT-BEV intravitreal bevacizumab, IVT-RAN intravitreal ranibizumab, SEM standard error of the mean.

The mean time in range for the three anti-VEGF agents according to three BCVA letter score thresholds, and unadjusted for baseline BCVA, are shown in Fig. 2A and Supplementary Fig. 1. As starting with higher VA makes it easier to spend time at or above a particular threshold, time in range was dependant on baseline BCVA. For participants with a baseline BCVA letter score ≥69 (approximate Snellen equivalent 20/40 or better), differences between the three anti-VEGF agents were only observed at the highest BCVA threshold (Fig. 2B).

When adjusted for baseline BCVA values, for threshold letter scores of ≥59, ≥69, and ≥79 (approximate Snellen equivalents of 20/63, 20/40, and 20/25 or better, respectively), time in range in the first 52 weeks of IVT-AFL treatment was 48, 41, and 22 weeks, respectively (Table 1). This was compared with 46, 37, and 17 weeks of time in range for IVT-BEV, and 46, 38, and 20 weeks for IVT-RAN, respectively. For the BCVA letter score threshold of ≥69 (approximate Snellen equivalent 20/40 or better), the least squares mean difference in the time in range in the Day 0–364 analysis was 4.0 weeks longer with IVT-AFL compared with IVT-BEV (*p* = 0.002), and 3.6 weeks longer with IVT-AFL compared with IVT-RAN (*p* = 0.004). There were similar observations for the BCVA letter score threshold ≥79 (approximate Snellen equivalent 20/25 or better), with IVT-ALF being associated with 5.3 and 2.0 additional weeks within time in range compared with IVT-BEV and IVT-RAN, respectively. For the BCVA letter score threshold ≥59 (approximate Snellen equivalent 20/63 or better), smaller differences in least squares mean time in range were observed between the three anti-VEGF agents (Table 1). Similar trends were also observed in the Day 365–728 and Day 0–728 analyses (Table 2). For the BCVA letter score threshold of ≥69, the least squares mean difference in the time in range in the Day 365–728 analysis was 3.9 weeks longer with IVT-AFL compared with IVT-BEV (*p* = 0.011), and 2.4 weeks longer with IVT-AFL compared with IVT-RAN (*p* = 0.106).

**Table 1:** Baseline BCVA-adjusted time in range for letter scores ≥59, ≥69, and ≥79 (approximate Snellen equivalents of 20/63, 20/40, and 20/25 or better, respectively) in the three treatment groups for the Day 0–364 analysis and least squares mean differences (ANCOVA) between treatments.

Weeks	Day 0–364 analysis					
	Treatment effects least squares mean (95% CI)			Treatment difference least squares mean (95% CI)		
	IVT-AFL	IVT-BEV	IVT-RAN	IVT-AFL vs IVT-BEV	IVT-AFL vs IVT-RAN	IVT-BEV vs IVT-RAN
≥59 letter score (~20/63 or better)	47.9 (47.0, 48.9)	46.3 (45.1, 47.5)	45.7 (44.4, 47.0)	+1.6 (+0.3, +3.2)	+2.3 (+0.6, +3.9)	+0.6 (–1.1, 2.4)
*p* value				0.077	0.019	0.485
≥69 letter score (~20/40 or better)	41.2 (39.7, 42.8)	37.2 (35.5, 42.8)	37.6 (35.9, 39.3)	+4.0 (+1.7, +6.3)	+3.6 (+1.3, +5.9)	−0.4 (–2.9, +2.0)
*p* value				0.002	0.004	0.738
≥79 letter score (~20/25 or better)	22.0 (19.9, 24.1)	16.7 (14.7, 18.8)	20.0 (18.0, 22.0)	+5.3 (+2.4, +8.3)	+2.0 (–0.9, +4.9)	−3.3 (–6.2, –0.5)
*p* value				0.001	0.171	0.045

*ANCOVA* analysis of covariance, *BCVA* best-corrected visual acuity, *CI* confidence interval, *IVT-AFL* intravitreal aflibercept, *IVT-BEV* intravitreal bevacizumab, *IVT-RAN* intravitreal ranibizumab.

**Table 2:** ANCOVA analysis of the least squares mean differences in time above threshold for letter scores ≥59, ≥69, and ≥79 (approximate Snellen equivalents of 20/63, 20/40, and 20/25 or better, respectively) between the three treatment groups for the Day 365–728 and Day 0–728 analyses.

Weeks	Day 365–728 analysis					
	Treatment effects least squares mean (95% CI)			Treatment difference least squares mean (95% CI)		
	IVT-AFL	IVT-BEV	IVT-RAN	IVT-AFL vs IVT-BEV	IVT-AFL vs IVT-RAN	IVT-BEV vs IVT-RAN
≥59 letter score (~20/63 or better)	48.9 (47.7, 50.2)	47.4 (45.9, 48.9)	48.1 (46.8, 49.5)	+1.6 (–0.4, 3.5)	+0.8 (–1.0, 2.6)	–0.7 (–2.8, 1.3)
*p* value				0.365	0.778	0.778
≥69 letter score (~20/40 or better)	45.2 (43.6, 46.9)	41.4 (39.4, 43.4)	42.8 (41.0, 44.6)	+3.9 (1.3, 6.5)	+2.4 (0.0, 4.9)	–1.5 (–4.1, 1.2)
*p* value				0.011	0.106	0.287
≥79 letter score (~20/25 or better)	30.6 (28.0, 33.2)	24.2 (21.6, 26.9)	28.3 (25.8, 30.9)	+6.3 (2.6, 10.1)	+2.3 (–1.4, 5.9)	–4.1 (–7.8, –0.4)
*p* value				0.003	0.221	0.059

Weeks	Day 0–728 analysis					
	Treatment effects least squares mean (95% CI)			Treatment difference least squares mean (95% CI)		
	IVT-AFL	IVT-BEV	IVT-RAN	IVT-AFL vs IVT-BEV	IVT-AFL vs IVT-RAN	IVT-BEV vs IVT-RAN
≥59 letter score (~20/63 or better)	97.0 (95.0, 99.1)	94.0 (91.4, 96.6)	94.5 (92.2, 96.9)	+3.1 (–0.2, 6.3)	+2.5 (–0.6, 5.6)	−0.5 (–4.0, 3.0)
*p* value				0.204	0.226	0.760
≥69 letter score (~20/40 or better)	86.6 (83.7, 89.6)	79.6 (76.1, 83.0)	81.2 (78.0, 84.4)	+7.1 (2.5, 11.6)	+5.4 (1.1, 9.7)	−1.7 (–6.3, 3.0)
*p* value				0.007	0.028	0.487
≥79 letter score (~20/25 or better)	52.7 (48.3, 57.1)	41.3 (36.8, 45.7)	48.6 (44.3, 52.9)	+11.4 (5.2, 17.7)	+4.1 (–2.0, 10.3)	−7.3 (–13.3, –1.1)
*p* value				0.001	0.187	0.042

*ANCOVA* analysis of covariance, *CI* confidence interval, *IVT-AFL* intravitreal aflibercept, *IVT-BEV* intravitreal bevacizumab, *IVT-RAN* intravitreal ranibizumab.

**Fig. 3:**
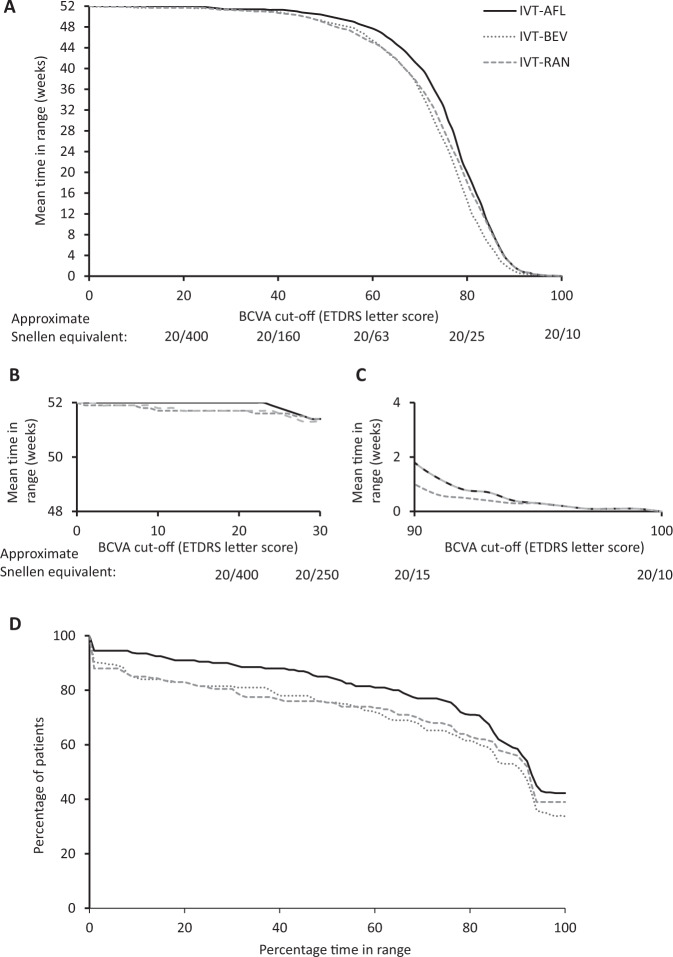
Mean time in range for intravitreal aflibercept, bevacizumab, and ranibizumab according to BCVA thresholds, and responder analysis. Mean time in range with intravitreal aflibercept, bevacizumab, and ranibizumab according to BCVA threshold in the Day 0–364 analysis (**A**) for all BCVA thresholds and focusing on BCVA letter score thresholds of (**B**) <30 (approximate Snellen equivalent 20/252) and (**C**) >90 (approximate Snellen equivalent 20/20). Responder analysis based on the proportion of participants who spent time in range above a BCVA letter score threshold of ≥69 (approximate Snellen equivalent 20/40 or better) for various cut-offs of the proportion of time in range within the treatment period in the Day 0–364 analysis (**D**). BCVA best-corrected visual acuity, ETDRS Early Treatment of Diabetic Retinopathy Study, IVT-AFL intravitreal aflibercept, IVT-BEV intravitreal bevacizumab, IVT-RAN intravitreal ranibizumab.

Mean time in range according to any BCVA letter score threshold from 0 (achieved 100% of the time) to 100 (achieved none of the time) for IVT-AFL, IVT-BEV, and IVT-RAN is shown in Fig. 3A for the Day 0–364 analysis (Supplementary Fig. 2 for the Day 365–728 and Day 0–728 analyses). For BCVA letter score thresholds between 30 and 92, the mean time in range was higher for IVT-AFL compared with IVT-BEV and IVT-RAN (Fig. 3A). Time in range does not differentiate between treatments below a BCVA letter score threshold of 30 (approximate Snellen equivalent 20/252; Fig. 3B; time in range >51 weeks), and above a letter score threshold of 92 (approximate Snellen equivalent 20/20; Fig. 3C; time in range <1 week).

In the Day 0–364 responder analysis, using a BCVA letter score threshold of ≥69 (approximate Snellen equivalent 20/40), 68% of participants treated with IVT-AFL had a time in range of ≥80% (i.e. for 41.6 weeks over Year 1), compared with 60% of participants treated with IVT-RAN and 55% treated with IVT-BEV (Fig. 3D). Responder analyses for the Day 365–728 and Day 0–728 analyses are shown in Supplementary Fig. 3.

## Discussion

In this post hoc analysis, we examined the novel concept of time in range, a potentially clinically relevant functional endpoint designed to examine response to treatment over time. This measure reflects the fluctuations in BCVA over time and, by incorporating a VA threshold that coincides with a patient’s ability to perform certain tasks such as driving, can be considered to indirectly reflect the impact of the disease on daily activities.

Using data from Protocol T to evaluate time in range in patients with DMO, we assessed the duration of time that a patient had BCVA letter scores of ≥69 (approximate Snellen equivalent 20/40 or better), chosen to align with the majority of global vision requirements for driving [9]. Among the 96.7% of patients who had ≥7 BCVA assessments in Year 1 of the study, the least squares mean time in range in the first year of treatment was 41.2 weeks with IVT-AFL, 4.0 weeks longer than IVT-BEV, and 3.6 weeks longer than IVT-RAN. Thresholds of BCVA letter scores of ≥59 (20/63) and ≥79 (20/25) were also evaluated. Between BCVA letter score thresholds of ≥30–<90 (approximate Snellen equivalent 20/250–20/15), patients receiving IVT-AFL had consistently better time in range than patients receiving IVT-BEV or IVT-RAN. Outside of this interval, where the sigmoid curve is horizontal, the time in range did not differentiate between the three anti-VEGF agents.

Although the use of time in range to monitor treatment efficacy has been used previously to assess blood glucose control in diabetic patients [6, 7] and international normalised ratios in patients receiving anticoagulants [10], a Medline search of the literature failed to identify a ‘time-in-range’ type of analysis in ophthalmic disease. Data from a post hoc analysis of time of vision loss of ≥5, ≥10, and ≥15 letters or of 20/40 or worse calculating patient-weeks of vision loss in patients with non-proliferative diabetic retinopathy reported a similar analysis using a reverse approach, essentially evaluating ‘time out of range’ [11].

Traditional clinical endpoints are often measured at a single point in time (for example, change in BCVA from baseline to Month 12; ETDRS letter score improvement ≥15 by Month 12). These methods do not, however, capture the variability in response to therapy that patients may have over the course of their treatment and consequently do not reflect potential impact on activities of daily living. In one example (Fig. 1A), two participants from Protocol T had similar changes in BCVA letter score from baseline to Month 12, but the proportion of time the clinically relevant BCVA letter score threshold of ≥69 (20/40 or better) was achieved differed by almost 30 weeks over the course of the first year of treatment. Furthermore, unlike the AUC calculation for the change in BCVA or the proportion of individuals with ≥15 letter gain (or other thresholds) over 1 or 2 years, the time in range calculates the number of weeks that the group achieves a threshold such as having the VA required to legally drive.

The Protocol T data used in exploring the time-in-range concept serve as a conduit for describing the application of the technique and demonstrate how this analysis assesses the consistency of treatment efficacy above a predefined threshold over time, rather than a static measurement at the end of the trial. This approach could be applied to either a treatment cohort to assess mean time in range or to an individual patient to monitor their treatment response. Time in range could also provide an additional or alternative approach for physicians when discussing clinical trial results with patients, by describing the number of weeks that an individual given a specific treatment might expect to experience VA above a certain threshold.

This time-in-range analysis shows similar trends in efficacy of the studied anti-VEGFs with respect to letter gains in BCVA over the course of the trial that reflect the prespecified primary outcome results reported for Protocol T. Also consistent with the findings from the Protocol T study, which showed that baseline BCVA was associated with BCVA gains at Month 12 [4], our analysis showed that baseline BCVA was associated with time-in-range outcomes.

The US Food and Drug Administration recognises the value of a patient-focused approach in drug development and is supportive of the development of novel, patient-focused endpoints [12]. The concept of glycaemic ‘time in range’ to assess variability in glycosylated haemoglobin levels in patients with diabetes is an established and valuable measure of treatment outcomes [13]. Its adoption has been facilitated by the availability of continuous glucose monitoring and the identification of glycaemic variability as an independent risk factor for diabetes complications [6, 7]. It remains to be determined whether time in range can have the same potential in routine clinical practice in DMO. Certainly, one immediate utility seems to be the use of BCVA time in range in randomised clinical trials that might enable a better understanding, for physicians and patients, of the consistency of treatment efficacy. Although not investigated in this analysis, we hypothesise that incorporating the time-in-range concept may facilitate physician–patient communications by providing additional ways in which physicians can describe the impact of treatment. It is hoped that future trials will investigate this further.

The concept of time in range is not limited to BCVA or patients with DMO; possible applications of the approach could be developed for OCT measurements, patient-reported outcomes, or for patients with other retinal diseases. Indeed, analyses from patients with DMO, including those in Protocol T, have shown that eyes with larger fluctuations in CST have worse VA compared with eyes with more stable anatomical outcomes [14]. Nor is the concept limited to application in a clinical trial setting. Data from routine clinical practice might provide an excellent repository for further retrospective analyses and validations subject to design biases, such as selection bias or those introduced by variable follow-up. Furthermore, with increasing availability of mobile medical devices, VA or OCT data that are generated via home monitoring devices [15, 16] may further enable prospective time-in-range analyses.

The approach used here to calculate time in range has certain limitations, especially when applied to a progressive disease and continuous endpoints. Although all available data were utilised for the time-in-range calculations, BCVA was not assessed daily and assumes patients to have the same BCVA value until a newer measurement was performed. It is, therefore, not 100% accurate and, as such, the data and interpretation should be treated with caution. In addition, as this method is reliant on repeat measurements, the frequency of these measurements may impact time-in-range results; increasing the number of measurements within a given timeframe results in a more sensitive time-in-range calculation. In this time-in-range analysis of Protocol T, a cut-off of ≥7 VA measurements was required within the first year, given that this corresponds with the dosing regimens of anti-VEGF agents and excluded 22/660 (3.3%) eyes with fewer measurements. As noted in our methodology, 2-year outcomes are provided as supplemental analyses, as more limitations must be considered for the data between 1 and 2 years given the variable follow-up schedule. For sensitivity analysis, we have shown stable results when considering different cut-off values from 0 to 100 letter scores other than 70. In addition, we have included the “Responder-type” analysis showing the percentage of responders when considering 0–100% time in range as a response. Additional sensitivity analysis for cut-offs of ≥6 or ≥8 measurements were not performed because similar numbers of eyes would have been excluded (17 [2.6%]) and 30 [4.5%]) as with the ≥7 cut-off, and any potential impact would have been minimal. When using time in range to compare IVT-AFL, IVT-BEV, and IVT-RAN, consistent results were obtained across BCVA letter score thresholds from ≥30 to <90; this observation minimises the possibility that the focus on the ≥69-letter score cut-off in the primary analysis produced anomalous findings.

In summary, time in range is a potentially clinically relevant visual endpoint to assess functional treatment outcomes over time beyond AUC outcomes presented previously in trials evaluating DMO.

## Summary

### What was known before

- Diabetic macular oedema (DMO), a common cause of impaired vision and blindness amongst diabetics, can develop at any stage of diabetic retinopathy and is known to wax and wane over time.
- In clinical trials, central VA is a surrogate outcome for every day vision-related functions such as reading, driving, watching television, and recognising faces; however, the extent of VA fluctuations that may impact these vision-related functions is not routinely measured in clinical trials.

### What this study adds

- In this analysis, we assessed how much time above various different VA thresholds can be achieved in DMO, focusing on a threshold of a best-corrected VA letter score ≥69 (approximate Snellen equivalent 20/40), chosen to align with the majority of global vision requirements for driving.
- DRCR.net Protocol T data comparing treatment response to three anti-VEGF agents were analysed post hoc and showed time in range above best-corrected VA letter score ≥69 was 41 weeks over Year 1 with intravitreal aflibercept, compared with 37 weeks for bevacizumab and 38 weeks for ranibizumab.
- Time in range may be another way to describe vision outcomes and potential impact on vision-related functions over time for patients with DMO and is a concept transferable to other outcomes and retinal conditions.

### Supplementary information

Supplementary material

## Data Availability

The protocol and public dataset for the DRCR Retina Network Protocol T trial (clinicaltrials.gov: NCT01627249) is available at https://public.jaeb.org/drcrnet/stdy.
