# Supplementary material for: Visual acuity time in range: a novel concept to describe consistency in treatment response in diabetic macular oedema
Source: Eye (Lond). 2023 Mar 28;37(16):3367–75. doi: 10.1038/s41433-023-02507-x (PMC10630493; doi:10.1038/s41433-023-02507-x)
Supplement: Supplementary file 1 — Supplementary material [file 41433_2023_2507_MOESM1_ESM.pdf]

A Novel Concept to Describe Consistency in Treatment Response in Diabetic Macular Oedema

Igor Kozak, Ian Pearce, Chui Ming Gemmy Cheung, Tobias Machewitz, George N. Lambrou, Daniel Molina, Lima Suleiman, Hossam Youssef, Neil M. Bressler

Supplemental Table 1. Frequency of BCVA assessments between Day 0–364 and Day 365–728

Supplemental Table 2. Participant baseline demographics and disease characteristics

Supplemental Fig. 1. Mean (±SEM) time in range for intravitreal aflibercept, bevacizumab, and ranibizumab according to BCVA letter score thresholds of ≥59, ≥69, and ≥79 (approximate Snellen equivalent of 20/63, 20/40, and 20/25 or better, respectively) in the Day 365–728 analysis for (**A**) the overall population and (**B**) according to baseline BCVA, and in the Day 0–728 analysis for (**C**) the overall population and (**D**) according to baseline BCVA

Supplemental Fig. 2. Mean time in range with intravitreal aflibercept, bevacizumab, and ranibizumab according to BCVA threshold in the (**A**) Day 365–728 and (**B**) Day 0–728 analyses

Supplemental Fig. 3. Responder analysis based on the proportion of participants who spent time in range above a BCVA letter score threshold of ≥69 (approximate Snellen equivalent of 20/40 or better) for various cut-offs of the proportion of time in range within the treatment period in the (**A**) Day 365–728 analysis and (**B**) Day 0–728 analysis

Supplemental Table 1. Frequency of BCVA assessments between Day 0–364 and Day 365–728.

	Day 0–364			Day 365–728		
	**IVT-AFL (*n* = 224)**	**IVT-BEV (*n* = 218)**	**IVT-RAN (*n* = 218)**	**IVT-AFL (*n* = 224)**	**IVT-BEV (*n* = 218)**	**IVT-RAN (*n* = 218)**
**Number of BCVA assessments**						
Mean±SD	12.5±2.4	12.6±2.1	12.4±2.3	8.6±3.8	8.6±3.9	8.6±3.9
Median	13.0	13.0	13.0	9.0	9.0	9.0
Range	1–16	1–17	1–16	0–18	0–15	0–15
**Total BCVA assessments, *n* (%)**						
0	0	0	0	16 (7.1)	13 (6.0)	18 (8.3)
1	3 (1.3)	1 (0.5)	1 (0.5)	1 (0.4)	4 (1.8)	4 (1.8)
2	3 (1.3)	1 (0.5)	1 (0.5)	5 (2.2)	5 (2.3)	2 (0.9)
3	0	1 (0.5)	1 (0.5)	2 (0.9)	9 (4.1)	4 (1.8)
4	1 (0.4)	0	1 (0.5)	11 (4.9)	13 (6.0)	10 (4.6)
5	0	1 (0.5)	2 (0.9)	16 (7.1)	12 (5.5)	7 (3.2)
6	1(0.4)	1 (0.5)	3 (1.4)	5 (2.2)	15 (6.9)	18 (8.3)
7	2 (0.9)	3 (1.4)	3 (1.4)	14 (6.3)	13 (6.0)	16 (7.3)
8	4 (1.8)	3 (1.4)	4 (1.8)	20 (8.9)	21 (9.6)	22 (10.1)
9	3 (1.3)	7 (3.2)	4 (1.8)	23 (10.3)	13 (6.0)	18 (8.3)
10	3 (1.3)	3 (1.4)	6 (2.8)	23 (10.3)	22 (10.1)	15 (6.9)
11	19 (8.5)	10 (4.6)	7 (3.2)	31 (13.8)	27 (12.4)	26 (11.9)
12	23 (10.3)	31 (14.2)	35 (16.1)	40 (17.9)	28 (12.8)	31 (14.2)
13	81 (36.2)	90 (41.3)	90 (41.3)	12 (5.4)	17 (7.8)	22 (10.1)
14	77 (34.4)	61 (28.0)	56 (25.7)	2 (0.9)	4 (1.8)	3 (1.4)
15	3 (1.3)	2 (0.9)	3 (1.4)	2 (0.9)	2 (0.9)	2 (0.9)
16	1 (0.4)	2 (0.9)	1 (0.5)	0	0	0
17	0	1 (0.5)	0	0	0	0
18	0	0	0	1 (0.4)	0	0

BCVA best-corrected visual acuity, *IVT-AFL* intravitreal aflibercept, *IVT-BEV* intravitreal bevacizumab, *IVT-RAN* intravitreal ranibizumab, *SD* standard deviation.

Supplemental Table 2. Participant baseline demographics and disease characteristics.

	Day 0–364 analysis			Day 365–728 and 0–728 analysis			Overall Protocol T population		
	**IVT-AFL (*n* = 216)**	**IVT-BEV (*n* = 213)**	**IVT-RAN (*n* = 209)**	**IVT-AFL (*n* = 200)**	**IVT-BEV (*n* = 187)**	**IVT-RAN (*n* = 188)**	**IVT-AFL (*n* = 224)**	**IVT-BEV (*n* = 218)**	**IVT-RAN (*n* = 218)**
**Age, mean±SD, years**	59±11	61±10	59±11	60±10	61±10	59±11	60±10	62±10	60±11
**Sex, *n* (%)**									
Female	104 (48)	101 (47)	90 (43)	93 (47)	92 (49)	81 (43)	110 (49)	103 (47)	94 (43)
**Race, *n* (%)**									
American Indian/Alaskan Native	2 (<1)	0	0	2 (1)	0	0	2 (<1)	0	0
Asian	2 (<1)	2 (<1)	3 (1)	2 (1)	2 (<1)	2 (<1)	2 (<1)	2 (<1)	4 (2)
Black/African American	29 (13)	35 (16)	35 (17)	27 (14)	31 (17)	32 (17)	32 (14)	37 (17)	37 (17)
More than one race	6 (3)	1 (<1)	1 (<1)	6 (3)	1 (<1)	1 (<1)	6 (3)	1 (<1)	1 (<1)
Native Hawaiian/Other Pacific Islander	2 (<1)	2 (<1)	0	1 (<1)	1 (<1)	0	2 (<1)	2 (<1)	0
Unknown/not reported	5 (2)	8 (34)	10 (45)	5 (3)	7 (4)	9 (5)	6 (3)	8 (4)	10 (5)
White	170 (79)	165 (78)	160 (77)	157 (79)	145 (78)	144 (77)	174 (78)	168 (77)	166 (76)
**Duration of diabetes, mean±SD, years**	16±11	17±10	17±10	16±11	17±11	17±10	16±11	17±10	17±10
**Type of diabetes, *n* (%)**									
Type 1	22 (10)	12 (6)	15 (7)	22 (11)	10 (5)	15 (8)	22 (10)	12 (6)	16 (7)
Type 2	188 (87)	200 (94)	188 (90)	172 (86)	176 (94)	169 (90)	196 (88)	205 (94)	196 (90)
Unknown	6 (3)	1 (<1)	6 (3)	6 (3)	1 (<1)	4 (2)	6 (3)	1 (<1)	6 (3)
**DRSS, *n* (%)**									
Missing	0	2 (<1)	1 (<1)	0	1 (<1)	1 (<1)	0	2 (<1)	1 (<1)
Absent or minimal NPDR (level 10-20)	7 (3)	6 (3)	3 (1)	7 (4)	5 (3)	3 (2)	7 (3)	6 (3)	5 (2)
Mild to moderately severe NPDR (level 35, 43, 47)	143 (66)	129 (61)	143 (68)	131 (66)	113 (60)	127 (68)	150 (67)	132 (61)	145 (67)
Severe NPDR (level 53)	16 (7)	14 (7)	16 (8)	13 (7)	12 (6)	16 (9)	17 (8)	15 (7)	18 (8)
Prior PRP; without current PDR (level 60)	17 (8)	21 (10)	16 (8)	16 (8)	17 (9)	15 (8)	17 (8)	21 (10)	17 (8)
Mild to moderate PDR (level 61 and 65)	28 (13)	30 (14)	22 (11)	28 (14)	28 (15)	18 (10)	28 (13)	31 (14)	23 (11)
High-risk PDR (level 71 and 75)	2 (<1)	7 (3)	8 (4)	2 (1)	7 (4)	8 (4)	2 (<1)	7 (3)	9 (4)
Cannot grade	3 (1)	4 (2)	0	3 (2)	4 (2)	0	3 (1)	4 (2)	0
**HbA_{1c}, mean±SD, %**	8.05±1.75	8.01±1.64	8.13±1.76	8.03±1.75	7.94±1.57	8.21±1.77	8.06±1.76	8.00±1.64	8.17±1.78
**HbA_{1c}, *n* (%)**									
<7%	59 (27)	64 (30)	57 (27)	54 (27)	60 (32)	48 (26)	62 (28)	65 (30)	58 (27)
≥7%	152 (70)	149 (70)	151 (72)	142 (71)	127 (68)	140 (75)	157 (70)	153 (70)	159 (73)
**CRT, µm**									
Mean±SD	457±134	456±130	455±118	455±133	459±133	452±116	459±134	455±129	456±119
Median (range)	429 (290–1115)	419 (266–940)	432 (248–860)	427 (290–1115)	419 (266–940)	430 (280–860)	430 (290–1115)	419 (266–940)	433 (248–860)
**BCVA in study eye**									
Mean±SD ETDRS letter score	65.1±11.6	64.7±11.1	64.6±11.4	65.1±11.7	64.5±11.1	64.9±11.3	65.0±11.6	64.9±11.0	64.6±11.4
Approximate Snellen equivalent	20/50	20/50	20/50	20/50	20/50	20/50	20/50	20/50	20/50
Median (range) ETDRS letter score	69.0 (78–24)	68.0 (78–24)	68.0 (78–29)	69.0 (78–24)	68.0 (78–24)	68.5 (78–29)	69 (78–24)	68 (78–24)	68 (78–29)
Approximate Snellen equivalent	20/40 (20/32–20/320)	20/40 (20/32–20/320)	20/40 (20/32–20/250)	20/40 (20/32–20/320)	20/40 (20/32–20/320)	20/40 (20/32–20/250)	20/40 (20/32, 20/63)	20/40 (20/40, 20/63)	20/50 (20/40, 20/80)
BCVA 68–0 (20/40 or worse), *n* (%)	105 (49)	107 (50)	105 (50)	96 (48)	95 (51)	94 (50)	112 (50)*	107 (49)*	110 (50)*
BCVA 100–69 (better than 20/40), *n* (%)	111 (51)	106 (50)	104 (50)	104 (52)	92 (49)	94 (50)	112 (50)*	111 (51)*	108 (5)*

*As reported in Wells JA, Glassman AR, Ayala AR et al. Aflibercept, bevacizumab, or ranibizumab for diabetic macular oedema. N Engl J Med. 2015;372:1193-203 (20/50 or worse [letter score <69] and 20/40 or better [letter score ≥70]). *BCVA* best-corrected visual acuity, *CRT* central retinal thickness, *DRSS* Diabetic Retinopathy Severity Scale, *ETDRS* Early Treatment Diabetic Retinopathy Study, *HbA_{1c}* 3-month average of blood glucose levels based on glycated haemoglobin measurements, *IVT-AFL*, intravitreal aflibercept, *IVT-BEV* intravitreal bevacizumab, *IVT-RAN* intravitreal ranibizumab, *NPDR* non-proliferative diabetic retinopathy, *PDR* proliferative diabetic retinopathy, *SD*, standard deviation.

Supplemental Fig. 1. Mean (±SEM) time in range for intravitreal aflibercept, bevacizumab, and ranibizumab according to BCVA letter score thresholds of ≥59, ≥69, and ≥79 (approximate Snellen equivalent of 20/63, 20/40, and 20/25 or better, respectively) in the Day 365–728 analysis for (**A**) the overall population and (**B**) according to baseline BCVA, and in the Day 0–728 analysis for (**C**) the overall population and (**D**) according to baseline BCVA.

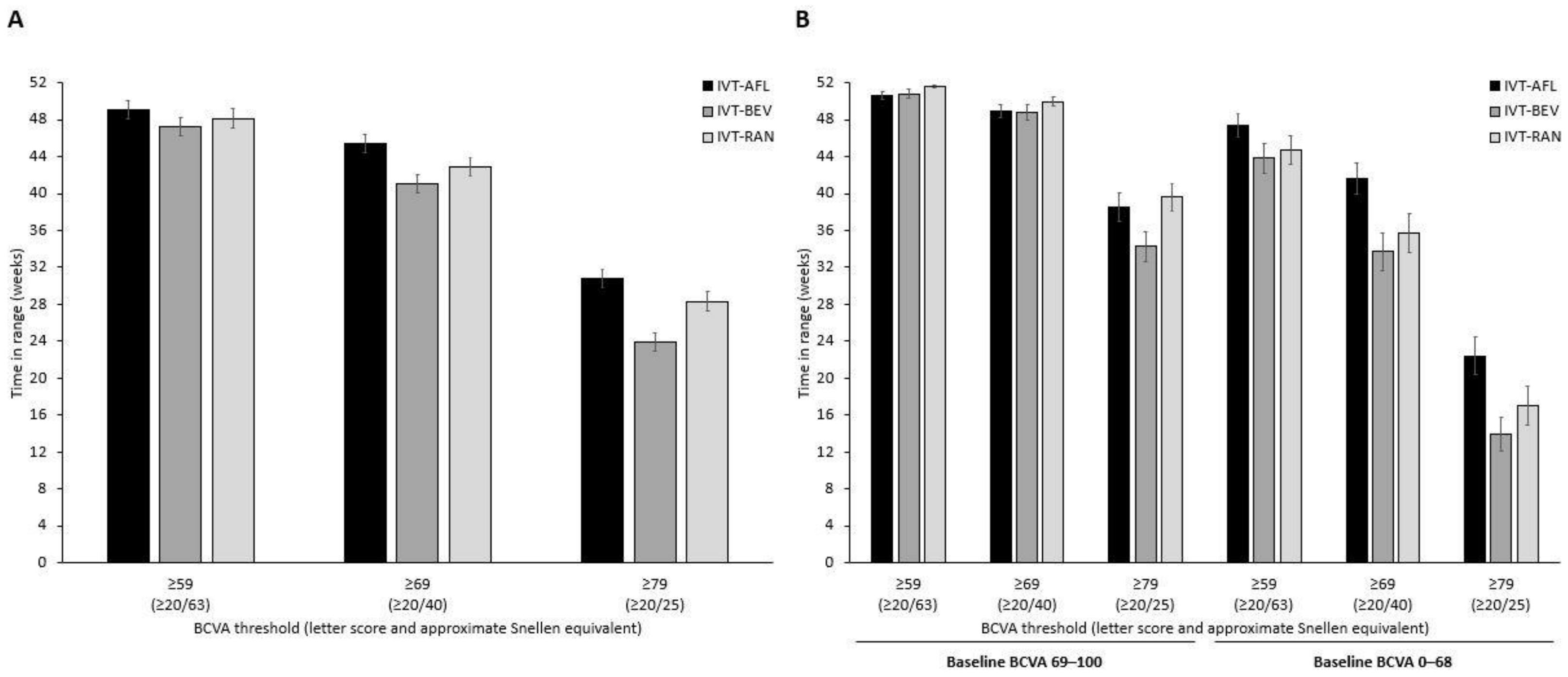

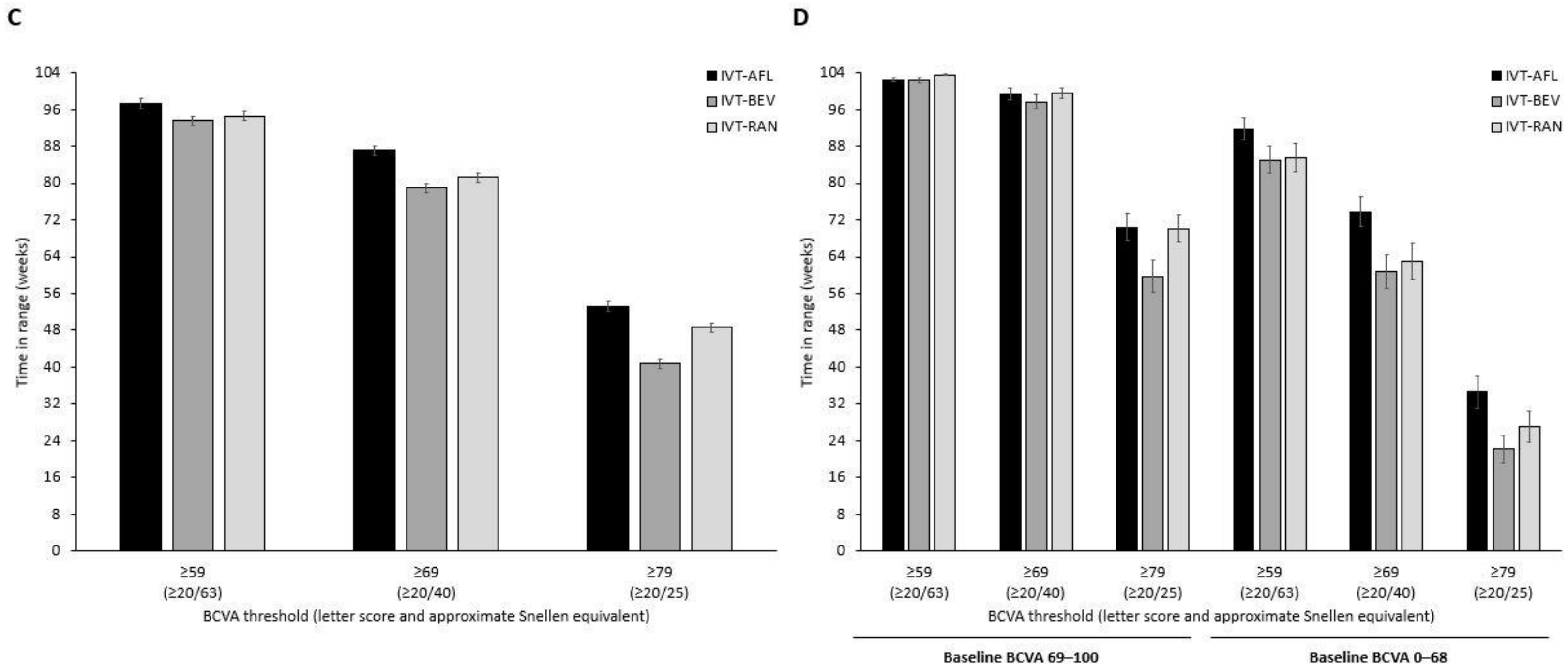

Of the 660 eyes included in Protocol T, 22 eyes were excluded from the Day 0–364 analysis because these participants had <7 BCVA assessments within this timeframe (approximately 1 VA assessment every 2 months); 85 eyes were excluded from the Day 0–728 and Day 365–728 analyses because participants had <4 assessments within Year 2.

BCVA best-corrected visual acuity, *IVT-AFL* intravitreal aflibercept, *IVT-BEV* intravitreal bevacizumab, *IVT-RAN* intravitreal ranibizumab, *SEM* standard error of the mean, *VA* visual acuity.

Supplemental Fig. 2. Mean time in range with intravitreal aflibercept, bevacizumab, and ranibizumab according to BCVA threshold in the (**A**) Day 365–728 and (**B**) Day 0–728 analyses.

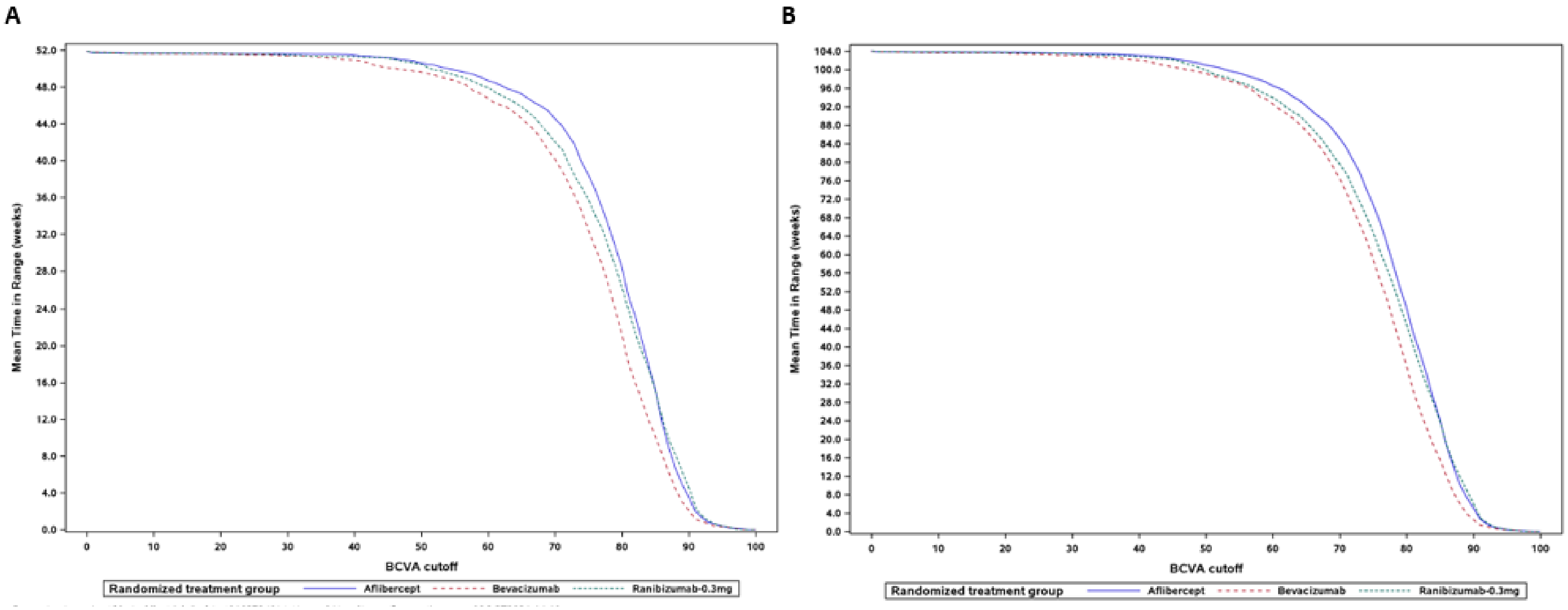

Of the 660 eyes included in Protocol T, 22 eyes were excluded from the Day 0–364 analysis because these participants had <7 BCVA assessments within this timeframe (approximately 1 VA assessment every 2 months); 85 eyes were excluded from the Day 0–728 and Day 365–728 analyses because participants had <4 assessments within Year 2.

BCVA best-corrected visual acuity, *VA* visual acuity.

Supplemental Fig. 3. Responder analysis based on the proportion of participants who spent time in range above a BCVA letter score threshold of ≥69 (approximate Snellen equivalent of 20/40 or better) for various cut-offs of the proportion of time in range within the treatment period in the (**A**) Day 365–728 analysis and (**B**) Day 0–728 analysis.

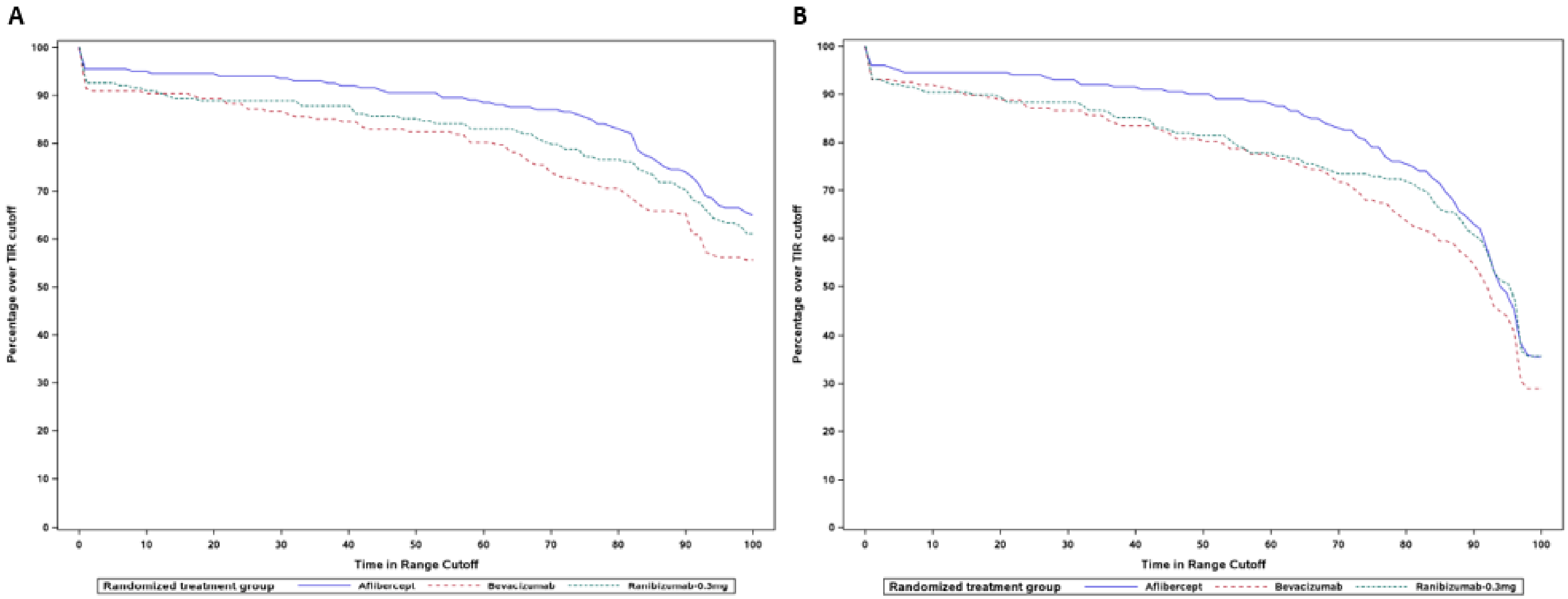

Of the 660 eyes included in Protocol T, 22 eyes were excluded from the Day 0–364 analysis because these participants had <7 BCVA assessments within this timeframe (approximately 1 VA assessment every 2 months); 85 eyes were excluded from the Day 0–728 and Day 365–728 analyses because participants had <4 assessments within Year 2.

BCVA best-corrected visual acuity, *TIR* time in range, *VA* visual acuity.
